# Evaluation of the safety and efficacy of a novel Anatomical classification and dUal anchoRing theory to Optimize the tavR strategy for pure severe Aortic regurgitation (AURORA): a prospective cohort study

**DOI:** 10.1186/s12872-022-02883-4

**Published:** 2022-10-15

**Authors:** Jing Yao, Zhi-Nan Lu, Thomas Modine, Hasan Jilaihawi, Nicolo Piazza, Yi-Da Tang, Ji-Zhe Xu, Xiao-Ping Peng, Hai-Ping Wang, Chuan-Bao Li, Hui Chen, Chang-Fu Liu, Zheng-Ming Jiang, Jie Li, Fei-Cheng Yu, Ke Han, Sheng Wang, Hui Huang, Guang-Yuan Song

**Affiliations:** 1 Interventional Center of Valvular Heart Disease, Beijing Anzhen Hospital, Capital Medical University, Beijing, 100029 China; 2 CHU de Bordeaux, Bordeaux, France; 3 NYU Langone Health, New York, USA; 4 McGill University Health Center, Montreal and German Heart Centre, Munich, Germany; 5 Third Hospital of Peking University, Beijing, China; 6 The First Hospital of Lanzhou University, Lanzhou, China; 7 Department of Cardiology, The First Affiliated Hospital of Nanchang University, Nanchang, China; 8 Qingdao Fuwai Cardiovascular Hospital, Qingdao, China; 9 Qilu Hospital of Shandong University, Jinan, China; 10 Beijing Friendship Hospital, Capital Medical University, Beijing, China; 11 Department of Cardiology, The Sixth Medical Center, Chinese PLA General Hospital, Beijing, China; 12 First Affiliated Hospital of Zhengzhou University, Zhengzhou, China; 13 Guangdong Provincial People’s Hospital, Guangzhou, China; 14 Sir Run Run Shaw Hospital (SRRSH), Affiliated with the Zhejiang University School of Medicine, Hangzhou, China; 15 The First Affiliated Hospital of Xi’an Jiao-Tong University, Xi’an, China; 16 Fuwai Central China Cardiovascular Hospital, Zhengzhou, China; 17 Cardiovascular Institute, Heart Center, General Hospital of Ningxia Medical University, Yinchuan, China

**Keywords:** Aortic regurgitation, Anatomical characteristics, Computed tomography, Transcatheter aortic valve replacement

## Abstract

**Background:**

Success rate of transcatheter aortic valve replacement (TAVR) in aortic regurgitation (AR) patients is relatively low on account of the absence of calcified anchoring structures. Morphological classification and corresponding TAVR strategies for AR are lacking yet.

**Methods:**

The AURORA study is a prospective, multicenter, single-arm cohort study to evaluate the safety and efficacy of transfemoral TAVR for severe AR in patients with high or prohibitive risk for surgery. Patients who are ≥ 65 years and diagnosed with severe pure AR as defined by the Echocardiographic Core Laboratory will be consecutively enrolled for further multidetector computed tomography (MDCT) scanning and multiplanar analyses. Based on a new anatomical classification and dual anchoring theory, patients will be classified into 4 types according to the level of the anchoring area. Types 1, 2 and 3 (at least 2 anchoring areas) will undergo the TAVR procedure with a domestic Chinese self-expanding valve (VitaFlow Valve, MicroPort, Shanghai, China), whereas type 4 (0 or 1 anchoring area) patients will be considered unsuitable for TAVR and will receive medical treatment. Our goal is to recruit 100 patients to account for 10% missing data or loss of patients to follow-up. Procedural, 30-day, 6-month and 12-month outcomes will be assessed according to Valve Academic Research Consortium-3 criteria.

**Discussion:**

The AURORA study will establish a new AR anatomical classification based on dual anchoring theory through MDCT multiplanar measurement and assess the safety and efficacy of TAVR guided by this new classification and strategy in AR patients.

*Trial registration*: This Study was registered at Chinses Clinical Trial Registry. The registration number: ChiCTR2200055415; The date of registration: 9, January 2022; The URL of the registration: http://www.chictr.org.cn/showproj.aspx?proj=141209.

## Background

**Fig. 1:**
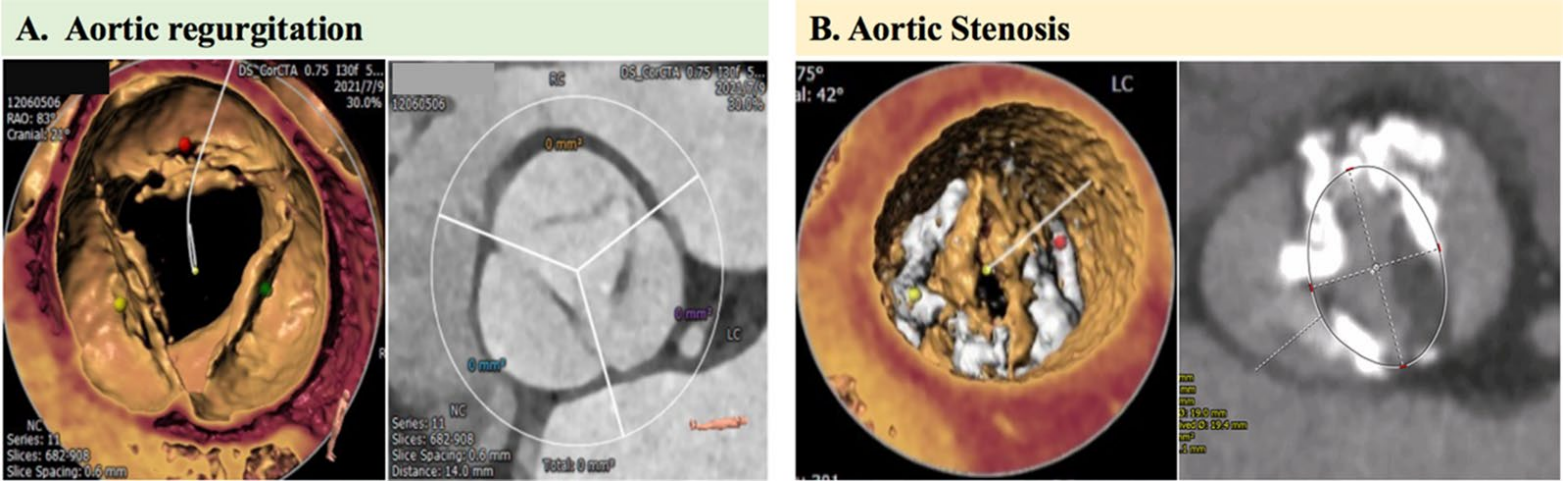
Unique aortic root morphological characteristics of pure aortic regurgitation compared to calcified aortic stenosis. Lack of a calcified landing level is a main challenge for TAVR in pure aortic regurgitation patients (**A**); Calcified spots of native aortic stenosis provide anchoring level for transcatheter heart valve in TAVR procedure (**B**). TAVR = transcatheter aortic valve replacement

Transcatheter aortic valve replacement (TAVR) has become the preferred treatment option in patients with severe aortic stenosis (AS) [1–3]. However, experience with applying TAVR in patients with pure native aortic regurgitation (AR) is limited. A recent epidemiological survey showed a prevalence of moderate to severe pure AR as high as 1.2% in China, and it was found to be the most frequent type of valvular dysfunction [4]. Even though patients with severe AR and left ventricular ejection fraction < 30% have an annual mortality risk of 20%, only 5% of the patients have an operation because of the high risk [5]. It thus becomes imperative to determine whether TAVR could be a feasible option in poor surgical candidates. However, AR is usually associated with abnormalities of the aortic cusp or root, without valvular calcification. These distinctive anatomical characteristics are extremely challenging to deal with when anchoring the device in TAVR (Fig. 1). Several registries have recently reported unsatisfactory outcomes of TAVR in patients with pure AR. The 30-day mortality rate has ranged from 9.3 to 23%. A total of 7.5–27.3% of patients required a new pacemaker; 0–4.7% had a stroke; 20.9–23% had moderate to severe paravalvular regurgitation; and 0–24.4% of patients needed a second transcatheter heart valve (THV) [6–10].

The presence of a double or multiple anchoring zones in the multiplane tract (from LVOT to ascending aorta) will significantly improves the stability of the prosthesis. A “TAVR-directed AR morphology classification” was proposed by our group. Based on this new classification and relevant procedure strategies, 32 cases of AR-TAVR using first-generation devices from China (The Venus A-Valve, Venus MedTech, China; VitaFlowTM transcatheter aortic valve, MicroPort®, Shanghai, China) have been performed before March 2021. The procedural success rate was 100% and the permanent pacemaker implantation rate was relatively low. These preliminary results are much better than many results using early-generation device reported by other centers. Therefore, we propose a prospective, multicenter, cohort study to evaluate the safety and efficacy of a novel **A**natomical classification and d**U**al ancho**R**ing theory to **O**ptimize the tav**R** strategy for pure severe **A**ortic regurgitation (AURORA).

The objectives of the proposed study are as follows:

- To construct an accurate morphological classification of aortic root anatomy in patients with severe pure AR to guide TAVR.
- To establish a novel double-anchoring strategy for transfemoral TAVR based on this new morphological classification.
- To evaluate the safety and effectiveness of transfemoral TAVR for patients with severe pure AR performed according to the double-anchoring theory and the new aortic root morphological classification.

## Methods

**Fig. 2:**
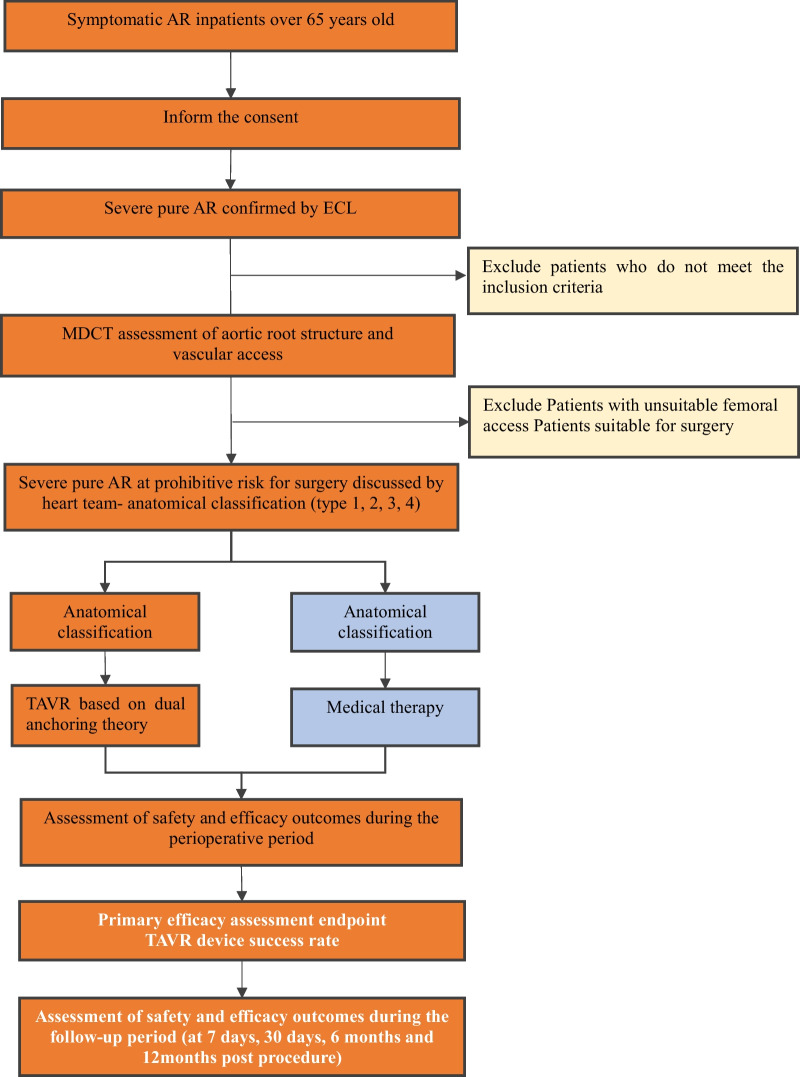
Study Flow Chart. *AR* aortic regurgitation, *ECL* echo core lab, *MDCT* multi-detector computed tomography, *TAVR* transcatheter aortic valve replacement

The AURORA study is a prospective, multicenter, single-arm, cohort study that will be conducted in 14 high-volume centers with specified expertise in TAVR. Patients with symptomatic pure severe AR confirmed by an Echocardiographic Core Laboratory who are ≥ 65 years will be consecutively enrolled for multidetector computed tomography (MDCT) scanning. After discussions with the heart team, the patients who are considered to have a prohibitive or high risk for surgery will be classified into 4 types: types 1, 2 and 3 patients who have more than 2 anchoring areas for THV will be treated with the TAVR procedure, whereas patients classified as type 4 who have only 1 area [either at the left ventricular outflow tract (LVOT), annulus, or ascending aorta (AA)] or no suitable area for anchoring will continue to receive medical treatment. Procedural and 30-day, 3-month, 6-month and 12-month outcomes will be assessed according to the Valve Academic Research Consortium-3 consensus (VARC-3 criteria). Enrollment is expected to be completed within 18 months. Institutional ethical approval will be requested in each center according to local regulations that comply with the principles outlined in the Declaration of Helsinki for research in human subjects. The study flow chart is depicted in Fig. 2.

### Study population

#### Inclusion criteria

Pure severe AR diagnosed by echocardiography; ≥ 65 years old; high surgical risk or surgical contraindications as discussed by the heart team; MDCT multiplanar with measurements; and a signed informed consent form.

#### Exclusion criteria

Patients with AS diagnosed by echocardiography; left ventricular ejection fraction < 20% and left ventricular end-diastolic diameter > 70 mm; poor medical compliance; participation in other interventional clinical trials within the past 30 days. The details of the inclusive and exclusive criteria are listed in Table 1.

**Table 1:** Study population

*Inclusion criteria*
1. Patients with pure severe AR diagnosed by echocardiography
2. > 65 years old
3. Patients with high surgical risk and surgical contraindications discussed by heart team
4. Multi-director computed tomography (MDCT) multiplanar measurements are complete
5. Informed consent form
*Exclusion criteria*
1. Patients with AS diagnosed by echocardiography
2. LVEF < 20% and LVEDD > 70 mm
3. Other contraindications for TAVR: Subjects with bacteremia or toxemia; active endocarditis; any intracardiac mass, left ventricular or atrial thrombus, vegetation diagnosed by echocardiography; severe mitral or tricuspid valve regurgitation; severe allergy to contrast agent, aspirin, heparin, thiazide, nickel-titanium memory alloy; other serious diseases that may reduce the life expectancy to less than 12 months; severe coagulation dysfunction; abdominal or thoracic aortic aneurysm; other conditions that the investigators determine are not suitable for this clinical study
4. Poor patient compliance, unable to complete the study as required
5. Have participated in other interventional clinical trials (within the past 30 days)

#### Protocol

The candidates will be screened and selected by our extended heart team, which includes an interventional cardiologist, a heart failure specialist, a cardiac imaging specialist, and a cardiac surgeon. The screening process includes an initial selection based on routine available measurements, dedicated confirmatory assessments (including echocardiography and MDCT), and final review by a central screening committee.

### Initial screening and informed consent

All patients with symptoms of heart failure and severe pure AR will be screened for eligibility to participate in the study by the local heart teams. The initial evaluation by the extended heart team is intended to evaluate the surgical risk based on routinely available information and to provide written informed consent to undergo the dedicated screening investigation and the subsequent morphological classification.

### Echocardiographic data analysis

The comprehensive transthoracic echocardiography or transesophageal echocardiography (if transthoracic echocardiography is suboptimal) data are uploaded to the Echocardiographic Core Laboratory for confirmation of the echocardiographic eligibility criteria. According to the 2017 American Society of Echocardiography guidelines [11], pure severe native AR is defined as severe AR without AS. The diagnosis of severe AR was based on the following criteria: (1) vena contracta width > 6 mm; (2) pressure half-time < 200 ms; (3) effective regurgitant orifice area ≥ 30 mm^2^; (4) regurgitant volume ≥ 60 ml/beats; and (5) dilation of the left ventricle.

### Multidetector computed tomography acquisition and analyses

#### Multiplanar analyses for anatomical characteristic of aortic root

Sixty-four-slice CT scans will be performed, using 100–120 kV, and tube current is modified according to patient size. All MDCT scans will be electrocardiogram-gated, primarily incorporating retrospective gating to yield a systolic phase for annular measurements. MDCT DICOM data are analyzed prospectively for all patients. Curved multiplanar reconstruction analyses will be performed using software specifically customized for valve analysis (3mensio Valves TM, version 10.1; 3mensio Medical Imaging BV, Bilthoven, The Netherlands).

**Fig. 3:**
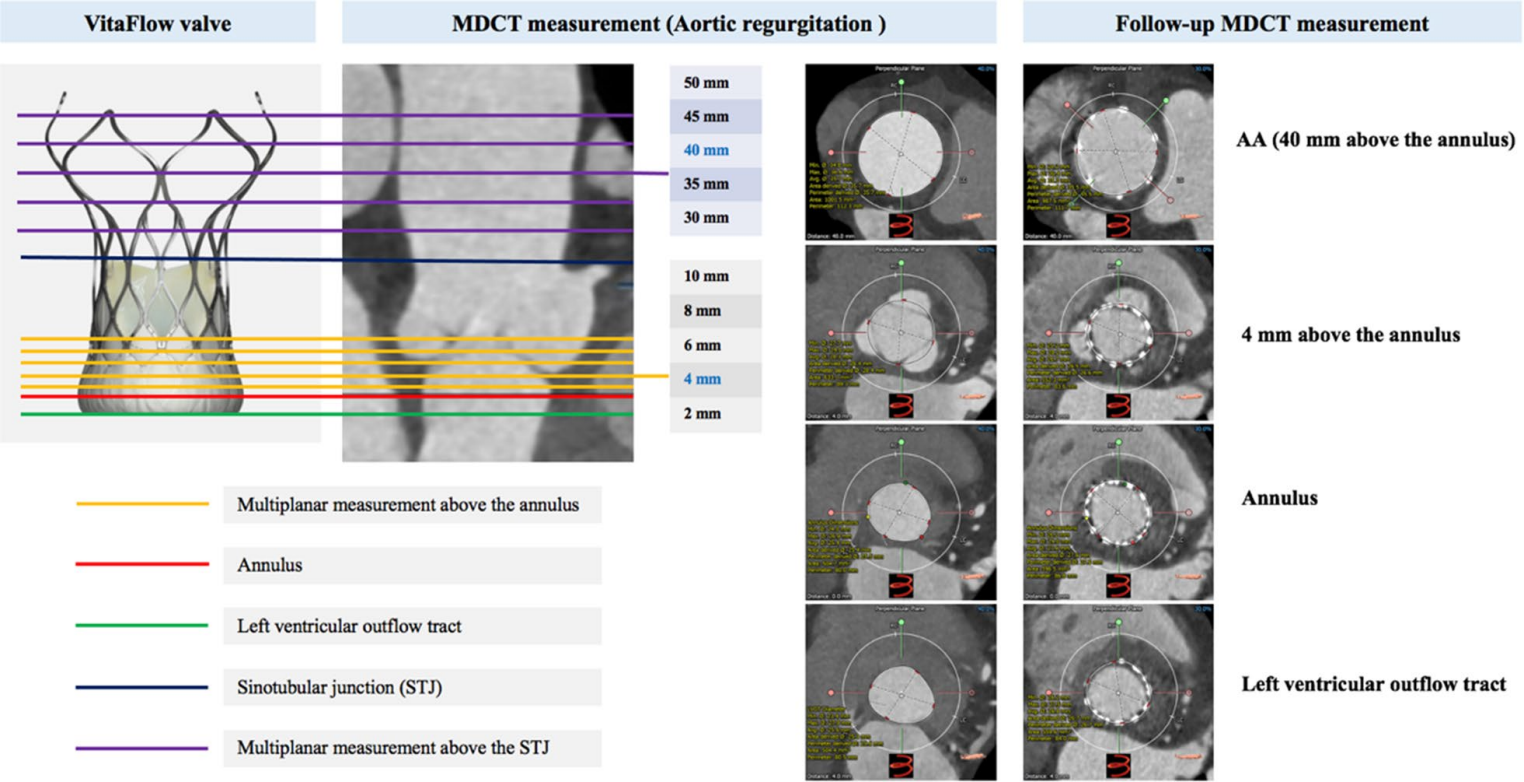
TAVR-specific AR dual-anchoring multiplanar measurements strategies. *TAVR* transcatheter aortic valve replacement, *AR* aortic regurgitation, *MDCT* multi-detector computed tomography, *AA* ascending aorta

A line is generated through the center point of the AA, aortic valve, annulus, and LVOT. The basal annular plane is defined as defined by the three basal attachment points of the aortic valve leaflets: the left-coronary cusp (LCC), the noncoronary cusp (NCC), and the right coronary cusp (RCC). A tracing of this annular plane provided maximal and minimal annulus diameters, area, and perimeter. LVOT, annulus, sinotubular junction (STJ), AA dimension 40 mm above the annulus, and maximal AA dimensions were measured routinely. Moreover, supra-annular and AA multiplanar dimensions (2, 4, 6, 8, 10, 30, 35, 40, 45, and 50 mm above the basal annular plane) will be also measured to identify the proper anchor levels for THV (Fig. 3).

### Multidetector computed tomography for vascular access suitability, including the aorta and iliofemoral axis

An aorta-ilio-femoral angiogram can be performed at the time of the preprocedural cardiac catheterization to facilitate vascular access evaluation, but this will not be a substitute for the MDCT.

### Transcatheter aortic valve replacement-directed aortic valve imaging: morphological classification

In a preliminary study, we found that the THV may be anchored at 3 areas in patients with pure AR: LVOT from − 4 to 0 mm below the aortic annulus, the annulus from 0 to 8 mm above the annulus, and the AA from the STJ to 40 mm above the annulus. THV anchoring was considered adequate if a perimeter oversizing index of > 10% was measured on the LVOT or the AA. A perimeter oversizing index of > 20% on a single annulus plane or of > 10% on every annulus area plane (0, 2, 4, 6, and 8 mm above the basal annular plane) is considered an adequate anchor on the annulus. If the THV can be anchored at more than two of the areas mentioned above with a considerable oversizing index rate, they are considered to have adequate anchoring stability for the TAVR procedure, which is termed the dual-anchoring theory**.**

Based on multiplanar measurements of aortic root anatomy and dual-anchoring theory, patients with AR will be classified into 4 types: Type 1: THV anchoring at the levels of the LVOT, the annulus, and the AA; type 2: THV anchoring at the levels of the annulus and the AA; type 3: THV anchoring at the levels of the annulus and the LVOT; type 4: THV anchoring at 1 level (either at the level of the LVOT, the annulus or the AA) or no anchoring at all.

**Fig. 4:**
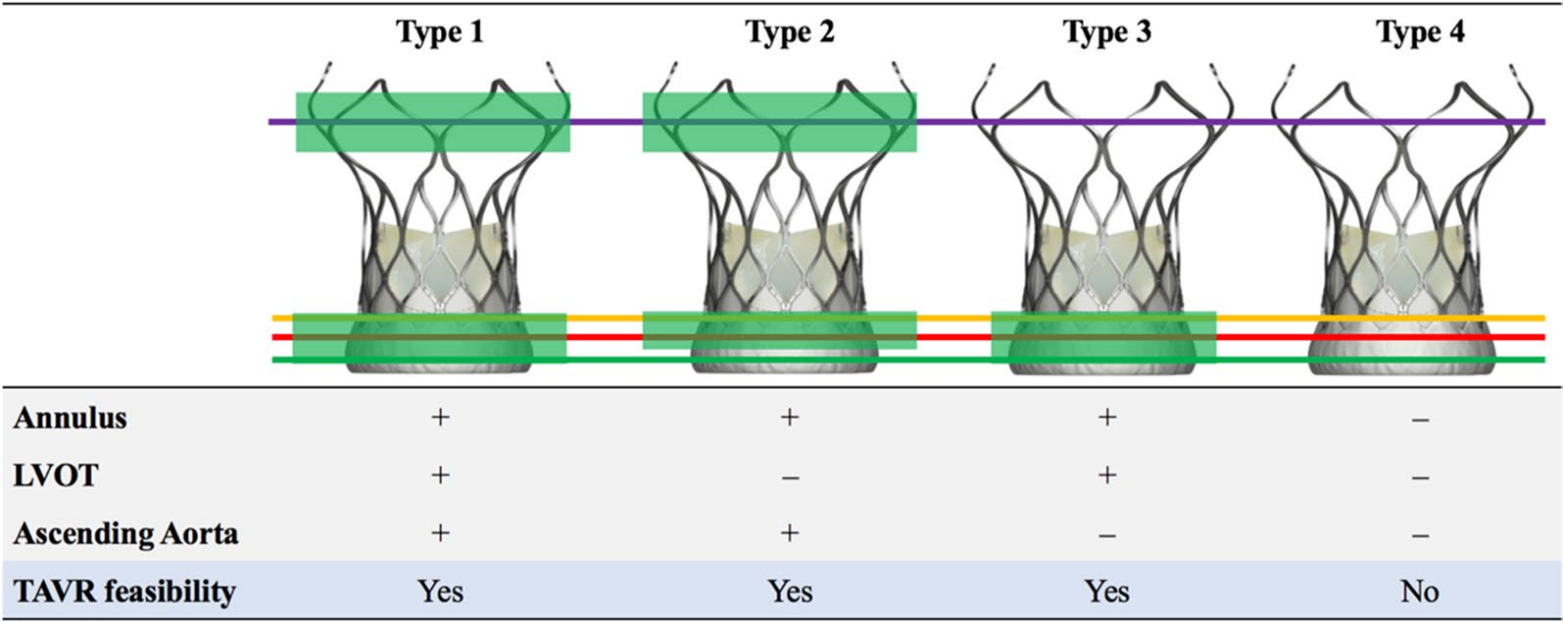
Schematic Diagram of TAVR-Directed AR Morphology Classification. Type 1: the LVOT, the annulus and supra-annular extending to ascending aorta can be anchored. Type 2: the LVOT cannot be anchored, but the annulus and ascending aorta can be anchored. Type 3: the annulus and LVOT can be anchored, but the ascending aorta cannot be anchored. Type 4: the LVOT, the annulus and supra-annular extending to ascending aorta cannot be anchored. THV anchoring was considered adequate if a perimeter oversizing index of > 10% was measured on LVOT or ascending aorta. Perimeter oversizing index of > 20% on single annulus plane or of > 10% on every annulus area planes (0, 2, 4, 6, 8 mm above the basal annular plane) is considered as anchoring adequate on annulus. Perimeter oversizing index was defined as [(device nominal perimeter)/(original perimeter measured by computed tomography)−1] * 100. AR Patients with type 1–3 can be candidates for TAVR, while type 4 is not suitable for TAVR treatment. *TAVR* transcatheter aortic valve replacement, *AR* aortic regurgitation

Patients with AR with types 1, 2 and 3 THV anchoring will be candidates for TAVR, whereas those with type 4 THV anchoring will be assigned to continue optimal guideline-directed medical treatment for heart failure (Fig. 4).

### Transcatheter aortic valve replacement procedure Devices

In this study, a domestic Chinese self-expanding TAVR device, the VitaFlow Valve (MicroPort, Shanghai, China) [12] will be used in patients with AR (Fig. 4).

### Procedures

All procedures will be performed with the patients given local or general anesthesia with intubation in the hybrid catheterization laboratory, under fluoroscopic guidance, by the multidisciplinary heart team. The transfemoral procedures will be performed as described previously [13, 14].

The following technical aspects will be investigated: (1) Releasing strategies: Rapid pacing (heart rate 180 beats/min) is recommended to decrease the regurgitation volume and systolic blood pressure. For patients classified as having type 2 anatomy, rapid pacing with 1 fast deployment will be used to improve the stability of the THVs. For patients with types 1 and 3 anatomical configurations, rapid pacing is used until two-thirds of the TAVR frame is released. Once the position is carefully checked with angiography, the THV is completely deployed. (2) Transcatheter aortic valve replacement-in-transcatheter aortic valve replacement technique: In patients in whom there is significant paravalvular regurgitation after the first THV deployment, it is suggested that a second THV should be implanted to reduce paravalvular regurgitation, malposition, or embolization of the first valve diving into the left ventricle.

### Study end points

The study end points will be defined according to VARC-3 criteria [15]. The primary efficacy end point is the device success rate. The second efficacy end point is the composite of technical success and improvement in AR severity after the procedure. Furthermore, we will investigate several additional secondary end points, including the rate of rehospitalization for heart failure, change in functional capacity as indicated by whether the patient is rated as the New York Heart Association functional class I or II, the 6-min walk distances, and the plasma concentration of N-terminal fragment brain natriuretic peptides (NT-proBNP) at 12 months compared with baseline. We will also assess myocardial remodeling by determining changes in the echocardiographic parameters of left ventricular remodeling at 12 months, as well as the quality of life utilizing the Kansas City Cardiomyopathy Questionnaire (KCCQ) score.

The safety end points include the major adverse cardiovascular and cerebrovascular events, which are defined as the composite end points of death, stroke, myocardial infarction, renal failure, arrhythmia, and conduction block occurring after the puncture, and reoperation for device- or procedure-related adverse events. All-cause mortality (30 days, 6 and 12 months after the procedure), cardiovascular mortality (30 days, 6 and 12 months after the procedure), and the post-procedure rehospitalization rate due to heart failure (30 days, 6 and 12 months after the procedure). All primary and secondary end points and the relationship of the event to the device and to the operation will be determined by an independent clinical event committee based on the prespecified definitions after review of the original medical documentation. The details of the study end points are summarized in Table 2. Details of the assessment performed during hospitalization and at each follow-up visit are depicted in Table 3. Follow-up visits are planned at 1 month, 6 months, and 12 months for all patients after discharge, irrespective of whether devices are implanted. Collection of routinely available clinical data will be discontinued only if explicitly request by the subject.

**Table 2:** Study endpoints

*Primary efficacy endpoint*
Device success rate (at 30 days) according to VARC-3 criteria Technical success (at exit from procedure room) Freedom from mortality Successful access, delivery of the device, and retrieval of the delivery system Correct positioning of a single prosthetic heart valve into the proper anatomical location Freedom from surgery or intervention related to the device* or to a major Vascular or access-related, or cardiac structural complication Freedom from mortality Freedom from surgery or intervention related to the device or to a major vascular or access-related or cardiac structural complication Intended performance of the valve‡ (mean gradient < 20 mmHg, peak velocity < 3 m/s, Doppler velocity index ≥ 0.25, and less than moderate aortic regurgitation)
*Secondary efficacy endpoints*
Technical success rate
The rate of patients with AR ≤ 2 + after surgery (7 days after procedure/before discharge, 30 days, 6 months and 12 months)
The rate of postoperative hospitalization for heart failure (7 days after procedure/before discharge, 30 days, 6 months and 12 months)
The rate of NYHA class I or II after procedure (7 days after procedure/before discharge, 30 days, 6 months and 12 months)
Improvement of 6MWD from baseline (baseline and 12 months after procedure)
BNP/NT-proBNP improvement from baseline (baseline and 12 months after procedure)
Changes of echocardiographic LVEDV from baseline (baseline and 12 months after procedure)
Improvement of quality of life as assessed by the Kansas City Cardiomyopathy Questionnaire (KCCQ) score (baseline and 12 months)
*Safety endpoints*
Incidence of MACCEs during the study
Defined as the composite endpoints of death, stroke, myocardial infarction, renal failure, arrhythmia, and conduction block occurring after the puncture approach, and reoperation for device- or surgery-related adverse events
All-cause mortality (30 days, 6 and 12 months after procedure)
All-cause deaths include cardiac deaths, non-cardiac deaths, and unexplained deaths
Cardiac mortality (30 days, 6 and 12 months after procedure)
Post-procedure rehospitalization rate due to heart failure (30 days, 6 and 12 months after procedure)

**Table 3:** Study data collection

Data collection	Baseline	Follow-up period				
		D1	D7/at discharge	D30	D180	D365
	− 7d	0d	0d	± 7d	± 15d	± 30d
Inform consent	X					
Demography	X					
Past history	X					
Vital sign	X		X	X	X	X
Physical Examination	X		X	X	X	X
History of angina	X		X	X	X	X
NYHA Functional Class	X		X	X	X	X
STS score	X					
Modified Rankin Scale (MRS) score	X		X	X	X	X
KCCQ score	X					X
6-min walk distance	X					X
ECG (18 leads)	X		X	X	X	X
TTE	X	X	X	X	X	X
MDCT	X				X	
CBC	X		X			
NT-proBNP	X					X
Coagulation Test	X		X			
Hepatic and renal function	X		X			
TNI/TNT	X		X			
Medication	X	X	X	X	X	X
Other treatments		X	X	X	X	X
AE/SAE Events	X	X	X	X	X	X

*NYHA* New York Heart Association, *STS* The Society of Thoracic Surgeon, *KCCQ* Kansas City Cardiomyopathy Questionnaire, *ECG* electrocardiography, *TTE* transthoracic echocardiography, *MDCT* multi-director computed tomography, *CBC* complete blood counts *NT-proBNP* N-terminal fragment brain natriuretic peptides, *TNT/TNI* troponins T and I, *AE/SAE* adverse events/severe adverse events

### Statistical analyses

Data will be expressed as mean ± standard deviation for continuous variables and compared using the unpaired Student *t*-test or the Mann–Whitney U test. Categorical variables will be reported as numbers with the relative percentage and compared using the χ^2^ or the Fisher exact test as appropriate.

A recent meta-analysis [1] has reported a device success rate of TAVR for AR of 80.4% (95% confidence interval, 72.2–88.6%). According to the results of our feasibility study using this novel anatomical classification and dual anchoring theory, the expected device success rate of this study is set at 90%. Consequently, a minimal number of 86 severe pure AR cases at high risk for surgery is needed to appropriately answer the primary objective of the study. Our goal is to recruit 100 patients to account for 10% missing data or loss of patients to follow-up. A p-value of < 0.05 will be considered significant. Statistical analyses will be performed using the SPSS software (version 24.0) (IBM Corp., Armonk, NY, USA).

## Discussion

To the best of our knowledge, this is the first prospective, multicenter cohort study to evaluate the safety and efficacy of TAVR for pure severe AR in a large sample of high-risk surgical patients. Moreover, our study will be the first to systematically analyze the anatomical characteristics of the aortic root in patients with pure native AR by MDCT multiplanar measurements. Based on this approach, we will propose a new anatomical classification and develop a novel dual anchoring theory with specific releasing strategies to improve the device success rate in TAVR, which will lead to significant breakthroughs in screening suitable patients for TAVR and improving the prognosis of patients with AR with a high risk for surgery.

Many existing studies have described the anatomical characteristics of the aortic root in patients with AS to guide the TAVR procedure [16]. However, a systematic analysis of aortic root anatomy in patients with AR is rare. Modern MDCT imaging approaches have the potential to accurately analyze the morphological characteristics of the aortic root in patients with AR, expanding from the LVOT to the AA, which may provide precise guidance for TAVR in high-risk surgical patients. Based on multiplanar analyses of aortic root morphology in patients with AR, we found that the THVs can be anchored not only in the annulus but also in other zones of the pathway from the LVOT to the AA. Dual anchoring areas will significantly improve the stability of the device in patients with pure AR. Therefore, we have developed a novel AR imaging classification to divide patients with AR into 4 types according to the anchoring levels. In patients with a type 1 anchoring level, THVs anchored from the LVOT–annulus area to the AA are the most stable. In type-2 patients, although the LVOT–annulus area is significantly dilated, the AA can provide a good anchoring level to improve device stability. In type-3 patients, the STJ and AA are severely dilated, and the LVOT–annulus levels play a major role in anchoring the THV, which may be more likely to result in the malposition of the THV.

Due to the significant differences in aortic root anatomy among the 3 types, we recommend different strategies for selecting the valve oversizing index and for deploying the valve. In patients with type 2, the THV should be released as rapidly as possible under rapid pacing (180 beats/min) until the AA anchoring works. For patients with types 1 and 3, we recommend deploying the THVs at the LVOT–annulus area in 2 stages as described previously. In these latter types, it is essential to ensure adequate anchoring without any significant perivalvular leakage before complete deployment.

In addition, we cannot ignore the fact that the large oversizing index may lead to compression of the conduction system by the valve frame and an increased risk of the need to implant a new pacemaker. In this study, we will use the VitaFlow device because this THV has a straight cylindrical shape with an outer skirt that may lead to increased friction between the prosthesis and the surrounding tissues and may help avoid displacement of the device. We speculate that the second-generation device with a recyclable system can be used in the higher releasing position to reduce the occurrence of abnormalities of the conduction system. However, the key breakthrough of TAVR for patients with AR is to design suitable devices based on the morphological classification and the dual anchoring theory.

## Data Availability

The datasets used and/or analyzed during the current study are available from the corresponding author on reasonable request.

## Abbreviations

AURORA: Anatomical classification and dUal anchoRing theory to Optimize the tavR strategy for pure severe Aortic regurgitation
TAVR: Transcatheter Aortic Valve Replacement
AS: Aortic Stenosis
AR: Aortic Regurgitation
THV: Transcatheter Heart Valve
MDCT: MultiDetector Computed Tomography
LVOT: Left Ventricular Outflow Tract
AA: Ascending Aorta
VARC-3: Valve Academic Research Consortium-3 consensus
LCC: Left Coronary Cusp
NCC: Non-Coronary Cusp
RCC: Right Coronary Cusp
STJ: SinoTubular Junction
NT-proBNP: N-terminal fragment Brain Natriuretic Peptides
KCCQ: Kansas City Cardiomyopathy Questionnaire
